# Evolution of Cement Pastes Blended with Ground Granulated Blast Furnace Slag (GGBFS) at Elevated Temperatures

**DOI:** 10.3390/ma19173804

**Published:** 2026-09-07

**Authors:** Michal Křištof, Marcin Sundin, Magdalena Rajczakowska, Andrea Jančíková, Simona Ravaszová, Hans Hedlund, Karel Dvořák, Andrzej Cwirzen

**Affiliations:** 1 Faculty of Civil Engineering, Brno University of Technology, Antonínská 548/1, 602 00 Brno, Czech Republic; andrea.jancikova@vut.cz (A.J.); simona.ravaszova@vut.cz (S.R.); karel.dvorak@vut.cz (K.D.); 2 Building Materials Group, Department of Civil, Environmental and Natural Resources Engineering, Luleå University of Technology, 97187 Luleå, Sweden; marcin.sundin@ltu.se (M.S.); magdalena.rajczakowska@ltu.se (M.R.); hans.hedlund@ltu.se (H.H.); andrzej.cwirzen@ltu.se (A.C.)

**Keywords:** blended cements, high temperature, microstructure evolution, in situ X-ray diffraction, melilite

## Abstract

**Highlights:**

- Real-time phase changes in blended cement pastes were tracked up to 1200 °C using an in situ XRD setup.
- Quartz-containing mixtures show earlier calcite and portlandite decomposition, driven by binder dilution and increased effective w/b ratio.
- Heating above 900 °C promotes melilite formation, supported by the fly ash content in CEM II/B-V R cement mixtures.

**Abstract:**

Mitigating structural failure and improving the fire safety of concrete infrastructure during severe thermal events depends critically on the high-temperature resilience of Portland cement paste. Given the increasing production of Portland blended cements, understanding their high-temperature behavior is crucial for ensuring the safety of building structures. This study investigates the effects of exposure to high temperatures (up to 1200 °C) on Portland cement pastes containing ground granulated blast furnace slag and quartz powder, focusing on their thermal stability and the chemical reactions occurring under these conditions. In situ X-ray diffraction (XRD) with a heating module was employed to observe real-time phase transformations as the temperature increased, supported by ex situ scanning electron microscopic analysis. The results showed changes in the mineralogical composition, with particular attention to the decomposition of calcium hydroxide and the formation of melilite above 900 °C. These transformations suggest thermal reactions between cement hydrate products (calcium silicates and aluminates) in the presence of slag and quartz powder. Mixtures containing quartz powder exhibited increased porosity and phase transformation shifts at lower temperatures, reflecting the combined effects of quartz addition, reduced reactive binder content, and an increased effective water-to-binder ratio. Notably, lower strength-grade cements containing fly ash (additional alumina source) show higher degrees of formation of calcium–aluminate silicate phases such as melilite upon heating. Compared to conventional studies, the novelty of this study lies in the use of an in situ experimental setup, which uniquely identifies the temperature thresholds of chemical changes and the formation of new phases such as melilite in cement–selected slag mixes, while also capturing their recrystallization upon cooling.

## 1. Introduction

Concrete is a composite material primarily consisting of a Portland cement-based binder, water, aggregates, and fillers. Its versatility, durability, and cost-effectiveness make it one of the most widely used construction materials [1,2]. In modern practice, Portland cement is often partially replaced with supplementary cementitious materials (SCMs) such as ground granulated blast furnace slag (GGBFS), ground limestone, or fly ash. These substitutions aim to reduce the CO_2_ footprint while also enhancing specific performance characteristics, including durability, chemical resistance, and mechanical strength [3,4,5].

Despite extensive research, the behavior of blended Portland cement under extreme conditions, particularly high temperatures, remains incompletely understood, especially under “in situ” scenarios. Advanced monitoring techniques have been employed to study thermal effects in concrete. Toropovs et al. [6] measured temperature, pressure, and moisture in high-performance concrete elements exposed to elevated temperatures using neutron radiography imaging, while Bao et al. [7] used optical fiber sensors to monitor temperature gradients and detect crack formation during heating. Stepkowska et al. [8] observed via XRD that the d-spacing of portlandite crystals increases with prolonged high-temperature exposure, particularly in weaker pastes. Partial carbonation replaces smaller OH^−^ ions with larger CO_3_^2−^ ions, lowering lattice energy, increasing thermal expansion, and reducing mechanical strength. Porous pastes are more susceptible to this transformation, which also decreases the decomposition temperature of portlandite.

The incorporation of SCMs, particularly slag and fly ash, introduces further chemical complexity. GGBFS, a byproduct of iron manufacturing [9], modifies the chemistry of calcium silicate hydrate (C–S–H) phases by lowering the calcium-to-silicon (C/S) ratio and increasing aluminum incorporation, which directly influences mechanical properties, durability, and thermal response. Gao et al. [10] demonstrated using in situ XRD that higher water-to-cement ratios promote mass loss and early cracking, fly ash reduces mass loss with minimal deformation, slag improves resistance to crack initiation but accelerates crack propagation once initiated, and higher sand content shifts shrinkage toward expansion due to quartz transformation. Thermal deterioration accelerates markedly above 400 °C. Moreover, slag-containing cements may release hydrogen sulfide (H_2_S) upon heating, a toxic and flammable gas, highlighting the importance of understanding chemical reactions during thermal exposure for safe handling [11].

Real-time monitoring of mineralogical and thermal changes during heating is critical for evaluating structural stability, phase transformations, and durability. Such monitoring allows the identification of potentially reversible transformations that are missed in post-cooling analyses. Hossain et al. [12] highlighted that pozzolanic reactions in high-strength volcanic ash concrete form additional C–S–H phases, improving mechanical performance and durability. Rashad [13] reported that in high-volume fly ash concretes, initial XRD analyses revealed quartz and mullite from fly ash, along with calcite and semi-crystalline C–S–H, while portlandite was fully consumed. Heating to 400 °C transformed C–S–H into the stronger tobermorite, enhancing compressive strength, particularly in the 70% fly ash mix. At 800–1000 °C, both C–S–H and tobermorite disappeared, leaving quartz and mullite, which helped retain some strength despite overall reductions.

In a separate investigation [14], XRD scans of high-volume slag (HVS) pastes exposed to elevated temperatures revealed distinct phase evolution depending on the presence of PC and the exposure level. At room temperature, samples showed a diffuse hump near 30° 2θ, indicating amorphous phases, with portlandite and ettringite only present in PC-containing mixes. With increasing temperature, portlandite decomposed around 500–600 °C, accompanied by strength loss due to cracking and volume changes (HVS). Beyond 600 °C, major phase transformations were observed: akermanite reappeared with significantly increased intensity, alongside the formation of gehlenite and merwinite, reflecting a shift from an amorphous to crystalline microstructure. At 800 °C, most HVS samples exhibited enhanced crystallization with strong akermanite, gehlenite, and merwinite peaks, whereas in a sample with 90% this transition occurred earlier, at 600 °C, aligning with its sharper strength degradation. Savva et al. [15] observed that heating affects strength, modulus of elasticity, rebound, and pulse velocity differently. Concretes with 10% micro-fine ash behaved similarly to pure OPC up to 750 °C, while pozzolanic concretes showed improved strength up to 300 °C but greater sensitivity at higher temperatures. Handoo et al. [16] further confirmed that exposure to 100–1000 °C causes gradual Ca(OH)_2_ loss in mortar, more pronounced at the surface, while carbonation remains minimal. Donatello et al. [17] also investigated cement paste with a high volume of fly ash. Binder pastes have shown that high-temperature exposure induces substantial chemical and microstructural transformations, particularly above 700 °C. Prior to heating, the paste contained calcite, minor gypsum, and fly ash phases, but no portlandite. At 800 °C, calcite and vaterite decomposed, while new crystalline phases such as wollastonite, gehlenite, diopside, and Fe-silicates formed, coinciding with strength gains and shrinkage. SEM–EDX analysis confirmed a transition from a gel-like microstructure at 600 °C to a molten vitreous phase at 800 °C, enriched in Ca and depleted in Al, indicating sintering and the crystallization of Ca– and Ca/Mg–silicate phases.

Based on these findings, it is hypothesized that the high-volume incorporation of slag and fly ash systematically alters the high-temperature phase evolution of cement pastes by driving the intensive crystallization of melilite-group minerals (specifically gehlenite and akermanite) within the 600–800 °C temperature range; furthermore, ultra-high replacement levels of slag (e.g., 90%) significantly lower the temperature threshold required to trigger this amorphous-to-crystalline microstructural transformation.

Cooling regimes also significantly affect post-fire performance. Rapid cooling (quenching) generally accelerates cracking, and strength loss compared to slow cooling, leading to more severe structural and mechanical degradation above 400 °C [18,19,20,21]. Consequently, both thermal exposure and cooling conditions play a critical role in the post-high-temperature behavior of concrete.

This study aims to integrate chemical, thermal, and real-time monitoring approaches to characterize structural and chemical changes in cement–slag blended pastes enriched with quartz powder during continuous heating up to 1200 °C. Specifically, this work evaluates how the type of cement, and the addition of quartz powder influences phase transformations and thermal stability under a fixed 50% slag replacement rate. By providing direct insights during the high-temperature exposure period, this investigation addresses existing data gaps to guide design strategies for thermally robust concrete mixtures.

## 2. Materials and Methods

### 2.1. Materials

This chapter lists the properties of the raw materials used in the experiment, namely Portland cement, ground granulated blast furnace slag, and quartz powder.

#### 2.1.1. Portland Cement

**Figure 1:**
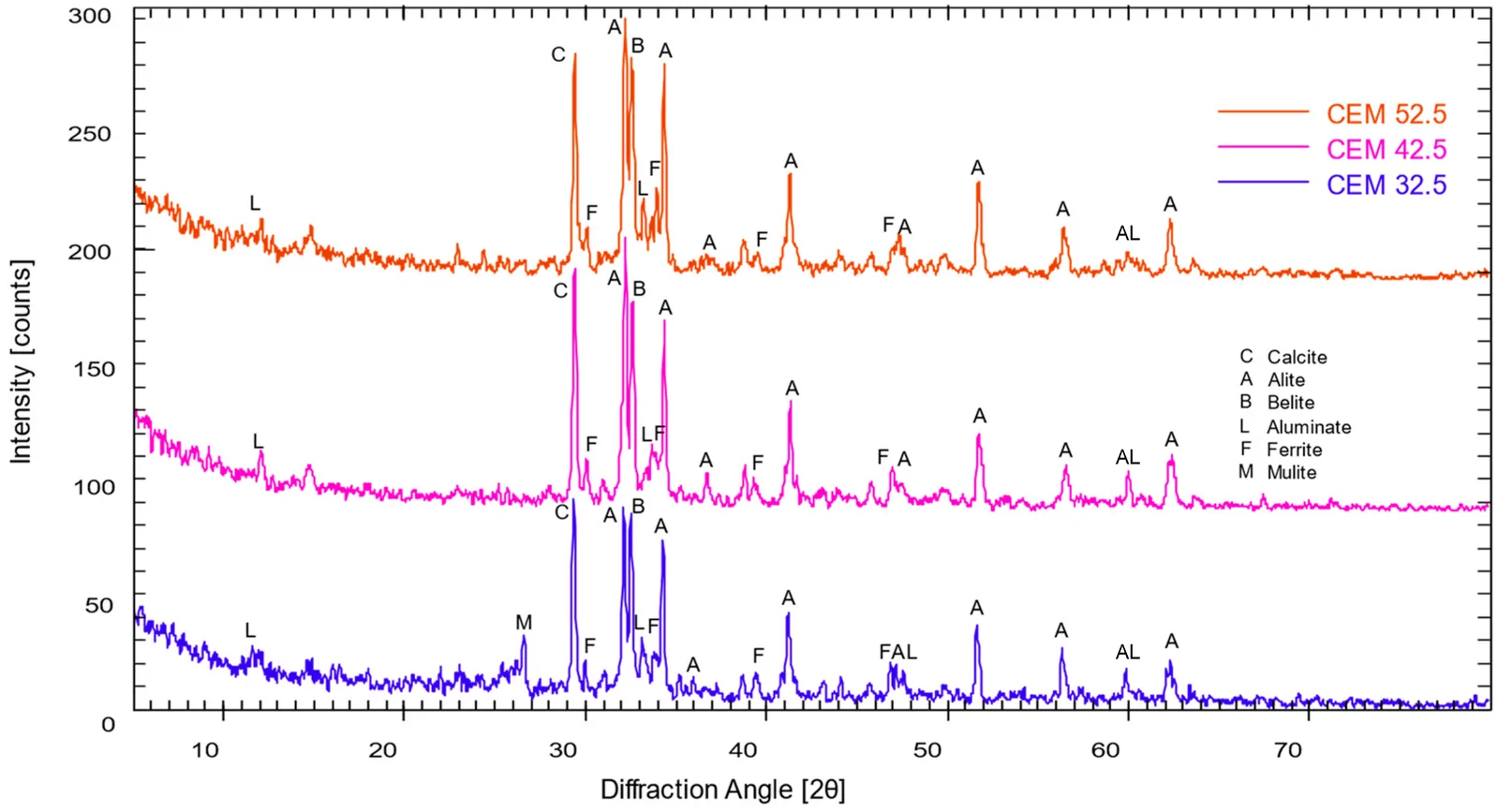
XRD diffractograms of the cements used in this study. Identified phases: C3S (A-Alite), C2S (B-Belite), C3A (L-Aluminate), C4AF (F-Ferrite), calcite (C), mullite (M).

The three different types of cement that were used in this study were provided by Cementa AB (Stockholm, Sweden) and CEMEX POLAND (Warsaw, Poland). They are specified according to EN 197-1 [22]: CEM II/B-V 32.5 R—HSR (fly ash-blended Portland cement); CEM I 42.5 N (Anläggningscement); and CEM I 52.5 R (Snabbcement Skövde). Their physical and chemical properties are summarized in Table 1. The XRD diffractograms are shown in Figure 1.

#### 2.1.2. Ground Granulated Blast Furnace Slag (GGBFS)

**Figure 2:**
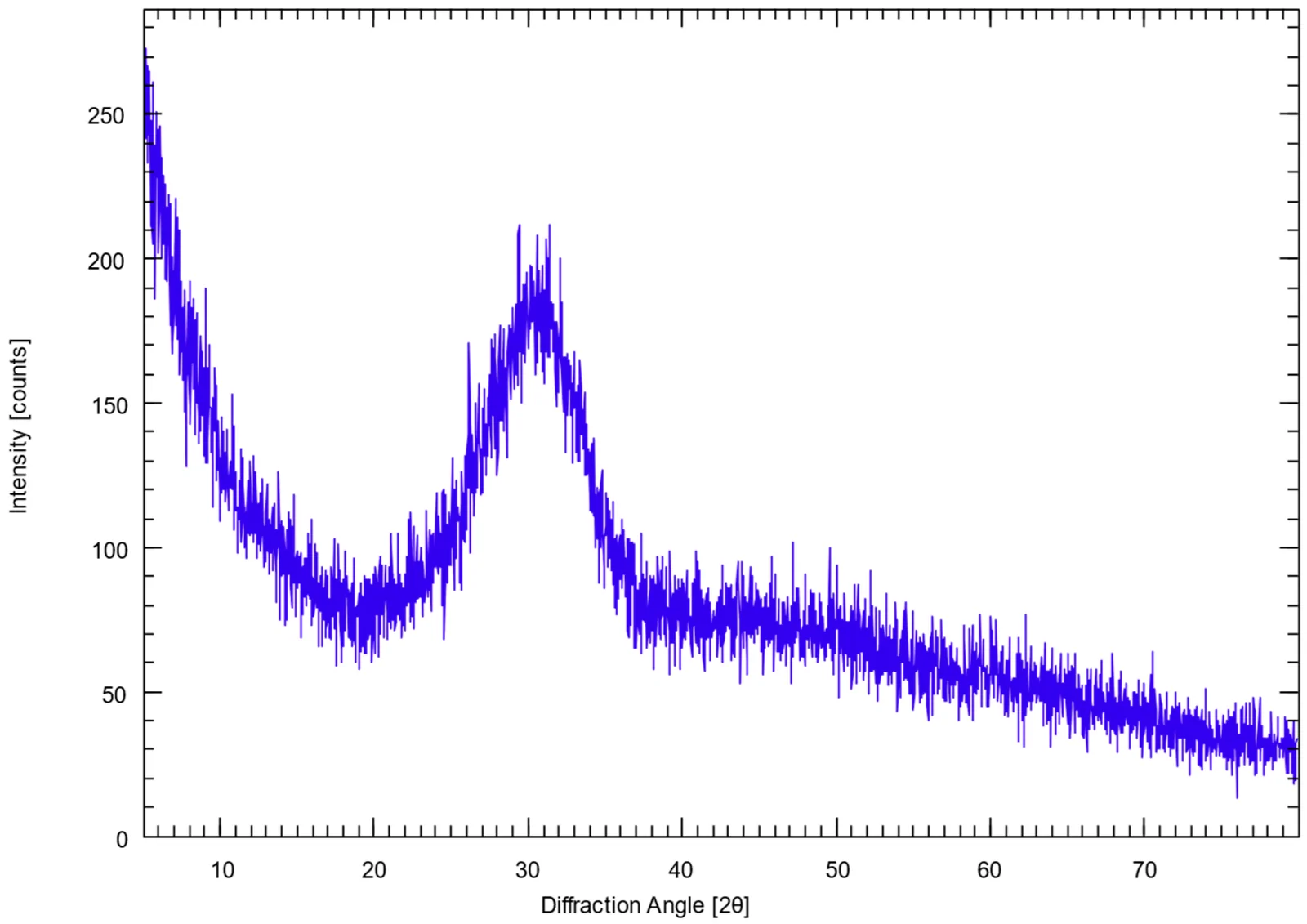
XRD diffractogram of GGBFS “Merit”.

The ground granulated blast furnace slag used in this study is available under the brand name “Merit,” and supplied by Swecem, Helsingborg, Sweden. The slag is provided with a Blaine fineness of 500 m^2^/kg and a particle density of 2950 kg/m^3^ according to manufacturer data. Its chemical composition is detailed in Table 2, and its XRD diffractogram is presented in Figure 2.

#### 2.1.3. Quartz Powder

**Figure 3:**
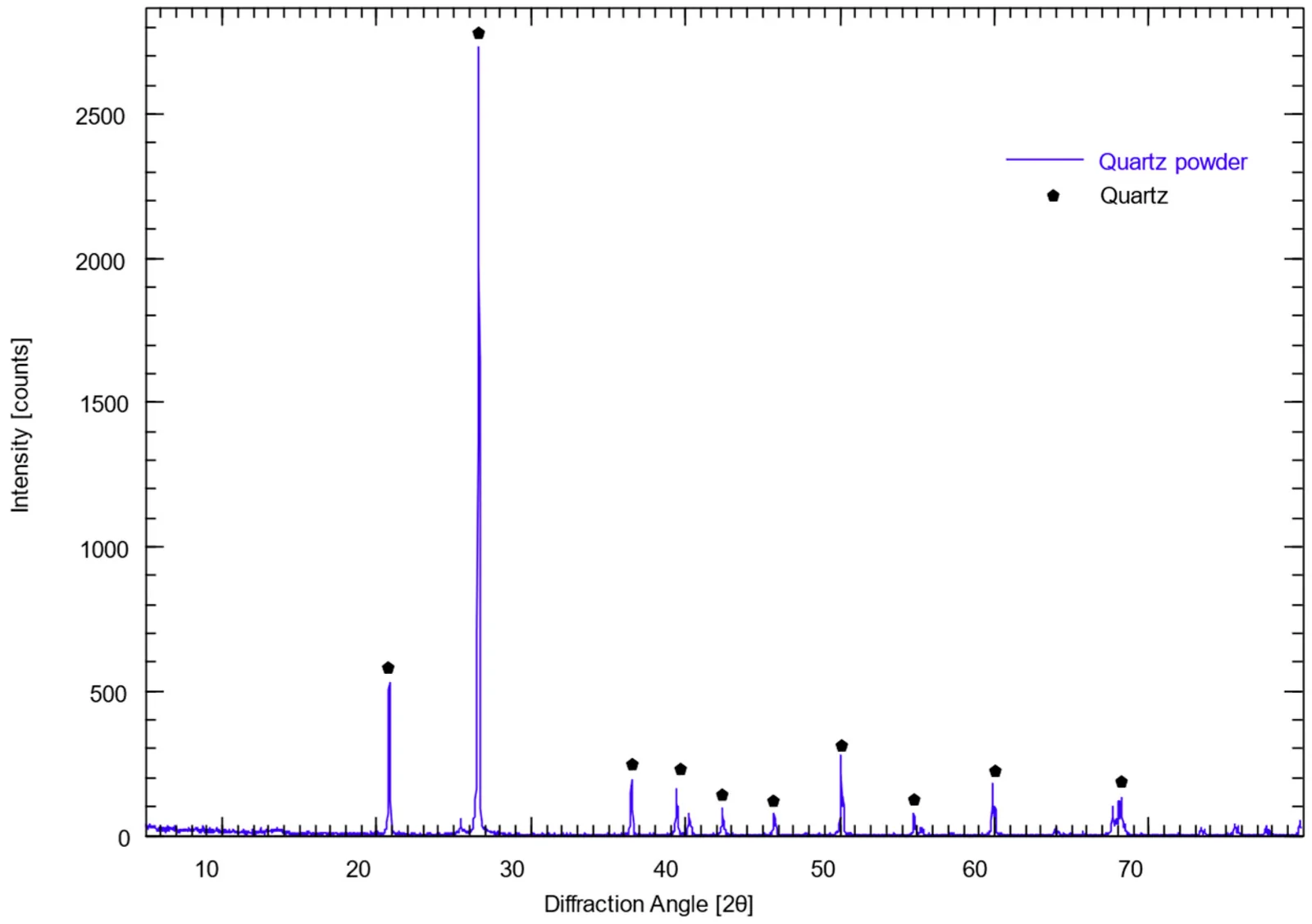
XRD diffractogram of Norquartz 45 powder. Identified phases: Quartz (Q).

The quartz powder Norquartz 45 was sourced from Sibelco, Habo, Sweden; see Table 3 and Figure 3.

### 2.2. Experimental Setup

#### 2.2.1. Preparation of Mixtures and Pastes

Paste samples were prepared according to the recommendations of RILEM TC-238 SCM [23]. The binder solids (cement, slag, and quartz filler) were first dry-mixed to give a unified powder, then mixed with distilled water at a water-to-solids ratio of 0.40; 80 g of solids were combined with 32 g of water; see Table 4. A vacuum laboratory mixer, EcoVac Bredent (Bredent GmbH & Co. KG, Senden, Germany), was used to mix the components at 390 rpm for 2 min to achieve a consistent mixture. After mixing, the mixture was poured into plastic molds, hermetically sealed, and kept at room temperature (21 °C) for 28 days. The mixtures created are referred to later in the text by their cement strength class, the abbreviation GGBFS and the proportion used 50.50. Mixtures containing quartz are additionally marked with the symbol “+Q” in the text. The use of cement strength classes is intended solely to simplify interpretation and does not in any way reflect a comparison of strength characteristics.

#### 2.2.2. Preparation of Samples for Analysis

To prepare samples for in situ XRD analysis, the pastes cured for 28 days were first crushed in a Retsch RS 200 disc mill (Retsch GmbH, Haan, Germany) for 10 s. Hydration was immediately stopped by washing the crushed material in isopropanol. The slurry was subsequently micronized using a McCrone Micronizing Mill (Retsch GmbH, Haan, Germany) for 150 s, also in isopropanol. The fine powder was gently dried at 30 °C in a laboratory dryer for 10 min and re-soaked in isopropanol to ensure complete hydration stoppage. Finally, the prepared powder was spread onto a thin platinum foil and mounted inside the high-temperature chamber (HTK) of the XRD instrument for continuous heating and real-time measurement up to 1200 °C.

For ex situ tests (XRD and SEM-EDX), paste samples were heated in a chamber furnace to 400 °C, 800 °C, and 1200 °C, and air-cooled. At least 3 samples per mix were prepared for each temperature. Prior to thermal loading, the samples were immersed in isopropanol for 7 days to remove all water. Following this, they were stored in a desiccator for 5 days. The post-firing samples from 400 °C, 800 °C, and 1200 °C were soaked into isopropanol again and stored in a desiccator for 7 days, impregnated with low-viscosity epoxy resin under vacuum, and polished with diamond sprays (particle sizes: 9 µm, 3 µm, and 1 µm) prior to SEM-EDX analysis [24]. For SEM-EDX, a cross-section of one sample was analysed. Additionally, the samples exposed to 400 °C and 800 °C were subjected to conventional room-temperature (ex situ) XRD measurements. The grinding and stopping of any potential chemical processes were carried out in the same way as for the in situ samples.

#### 2.2.3. Methods

The high-temperature XRD analysis was performed using a Panalytical Empyrean diffractometer (Malvern Panalytical B.V., Almelo, The Netherlands) with a high-temperature chamber Anton Paar HTK 2000N (Anton Paar GmbH, Graz, Austria) (platinum heating strip). The Θ-Θ reflection Bragg–Brentano para-focusing geometry device is equipped with a Cu anode (λ = 1.54184 Å), and programmable divergence slits a PIXcel3D detector (Malvern Panalytical B.V., Almelo, The Netherlands) with 255 active channels. The X-ray generator settings were 45 kV and 40 mA. The measured range was 6–60° with a step size between data points of 0.026° and a 101.5 s per step increment of the multi-point detector. The total measurement time for each sample was 19 min. The irradiated area was 100 mm^2^. The measurements were performed repeatedly in steps of 100 °C, between 100 °C and 1200 °C. The rate between each step was 30 °C/min. The initial and the final measurements were taken at 25 °C. The PROFEX 5.2.5 software was used to identify the individual phases. The COD database (2024 release) was used for the analysis of the diffraction patterns via a fundamental parameters approach [25]. Peak integrals were calculated via a peak integration module by selecting a specific angular range across a peak. No normalization was applied.

Due to the methodological limitations of the in situ high-temperature XRD setup, a reliable quantitative phase analysis could not be performed. Achieving a perfectly uniform dispersion of the cement paste particles on the platinum heating strip was technically challenging. Furthermore, because of the thin sample layer, the X-ray beam partially penetrated through to the underlying platinum substrate—particularly at high diffraction angles—leading to variations in the effectively irradiated sample volume. This effect was further compounded by severe thermal shrinkage and micro-cracking of the paste, which disrupted the required flat-sample geometry. Consequently, the analysis was restricted to qualitative phase identification and the evaluation of relative intensity variations via integral peak heights across the investigated temperature range.

An LAC LH15 furnace was used for high-temperature exposure of ex situ analyzed samples. The temperature rises to 400 °C in 30 min (13.33 °C/min), 800 °C in 175 min (4.57 °C/min) and 1200 °C in 290 min (4.14 °C/min). After reaching the target temperature, the temperature conditions were maintained for 1 h. Prepared paste samples were placed directly on the chamber floor without any spacing. After exposure, all samples were cooled down to room temperature within the chamber for about 12–18 h.

The scanning electron microscope (SEM) used for this analysis was a JSMIT100 instrument (JEOL Ltd., Akishima, Tokyo, Japan), equipped with a Bruker Quantax energy-dispersive spectrometry (EDX) system (Bruker Nano GmbH, Berlin, Germany). Magnifications of up to 500× were applied. Images were taken in backscattered electron (BSE) mode with an acceleration voltage of 15 kV, an electron beam current of 50 nA, and a chamber vacuum of 30 Pa. The chemical composition was determined using single-point measurements at 30 different locations across the cross-section.

**Figure 4:**
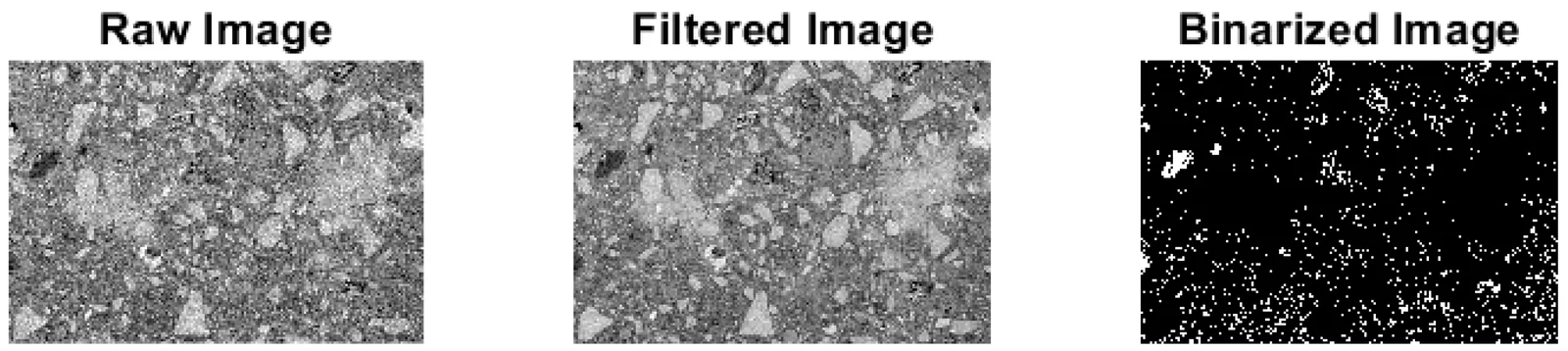
Example of cement paste image processing.

Matrix porosity was determined by analyzing backscattered electron (SEM-BSE) images. For each experimental condition, a single representative specimen was examined (*N* = 1). A total of 30 BSE images per specimen were acquired at 400× magnification. To focus the quantification strictly on matrix-level capillary porosity, images intersected by macro-cracks (on average, 2–3 images per sample batch) were excluded from the analysis. Image binarization was performed using the overflow method [26], defining porosity as the area fraction of porous pixels relative to the total image area (Figure 4). For specimens exposed to 1200 °C, where severe phase transformations and local melting altered the BSE contrast, global thresholding with manually adjusted limits based on local histogram minima was applied by a single operator to ensure segmentation consistency. Because individual SEM images obtained from a single specimen represent subsamples rather than independent statistical replicates, inferential statistics were omitted to avoid pseudoreplication. Instead, the results are presented using descriptive analysis, reporting mean values, standard deviations, and range distributions across the analyzed image fields.

#### 2.2.4. Use of Generative AI for Visual Abstract Creation

The Graphical Abstract was generated using Google Gemini to conceptualize and generate the initial layout and visual components. The input prompts were: illustrate the linear experimental progression: from raw material formulation, through in situ high-temperature XRD characterization, to the resulting microstructural transformation. The generated outputs were subsequently processed using Microsoft Designer for annotation, scaling, and formatting. All AI-generated content was manually reviewed by the authors to ensure accuracy and consistency with the experimental data.

## 3. Results and Discussion

### 3.1. High-Temperature In Situ XRD

**Figure 5:**
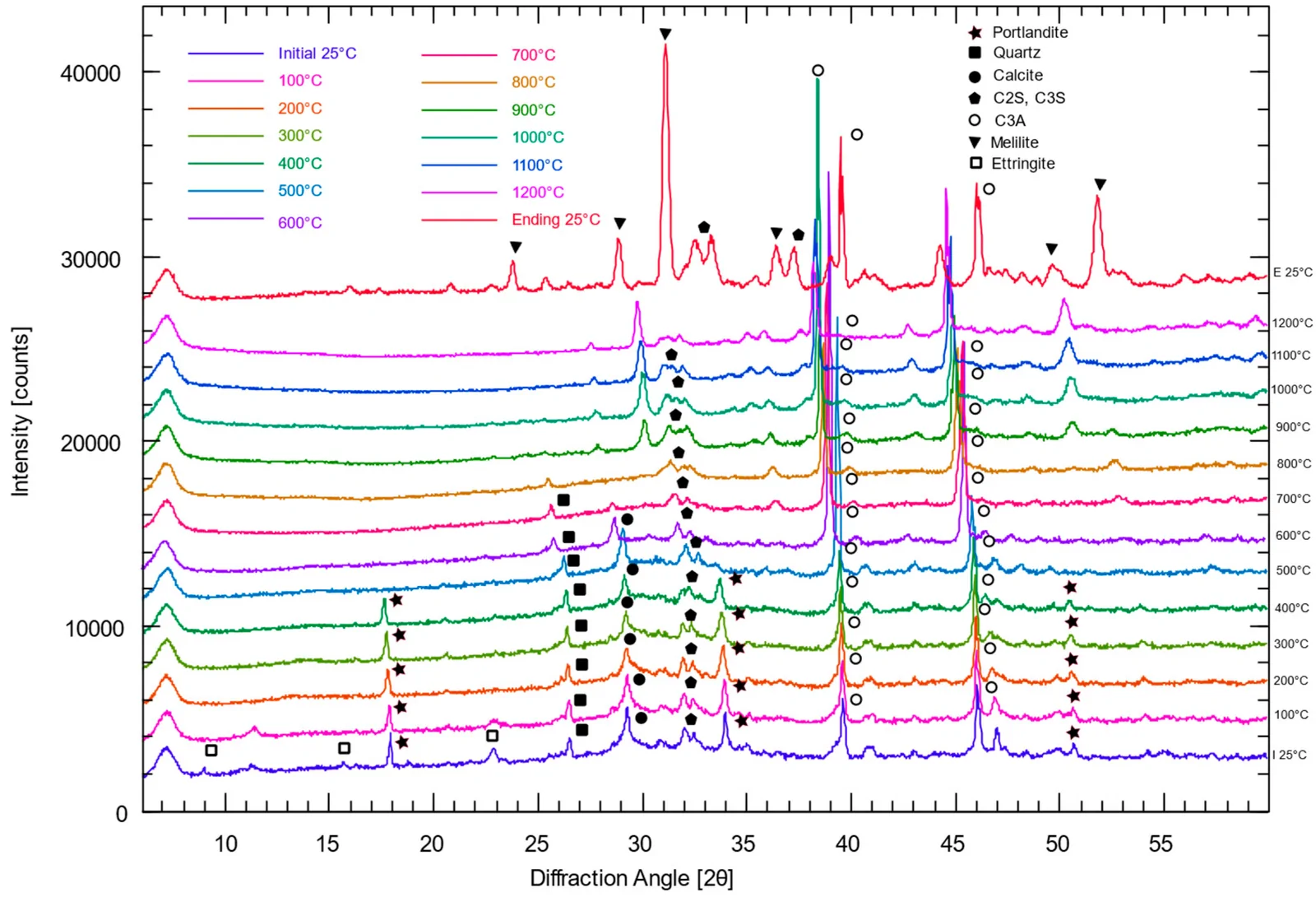
CEM 32.5 GGBFS 50.50 XRD diffractograms. Identified phases: portlandite (★), quartz (■), calcite (⬤), C2S C3S (⬟), C3A (○), melilite (▼), ettringite (□).

**Figure 6:**
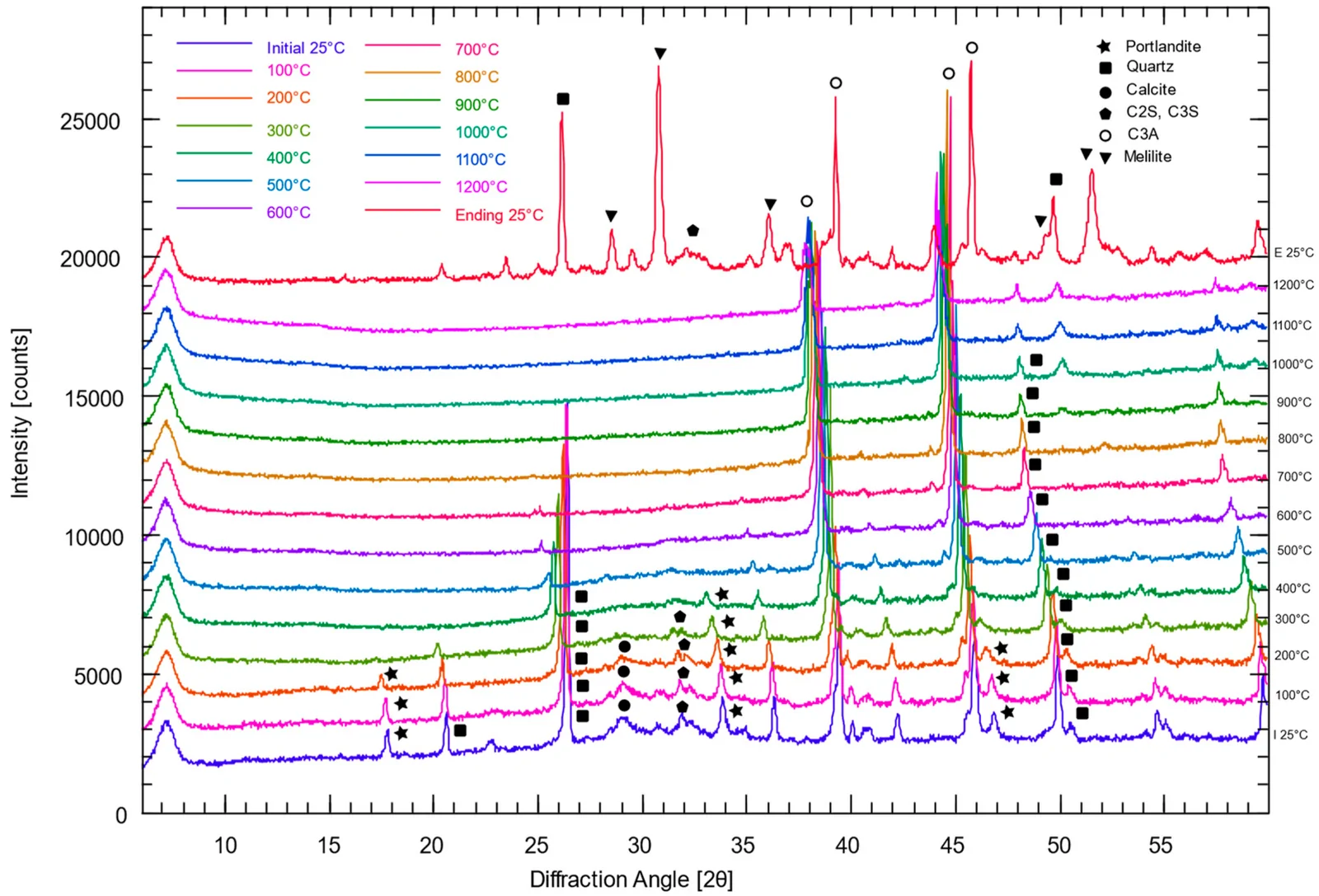
CEM 32.5 GGBFS 50.50 + Q XRD diffractograms. Identified phases: portlandite (★), quartz (■), calcite (⬤), C2S C3S (⬟), C3A (○), melilite (▼).

**Figure 7:**
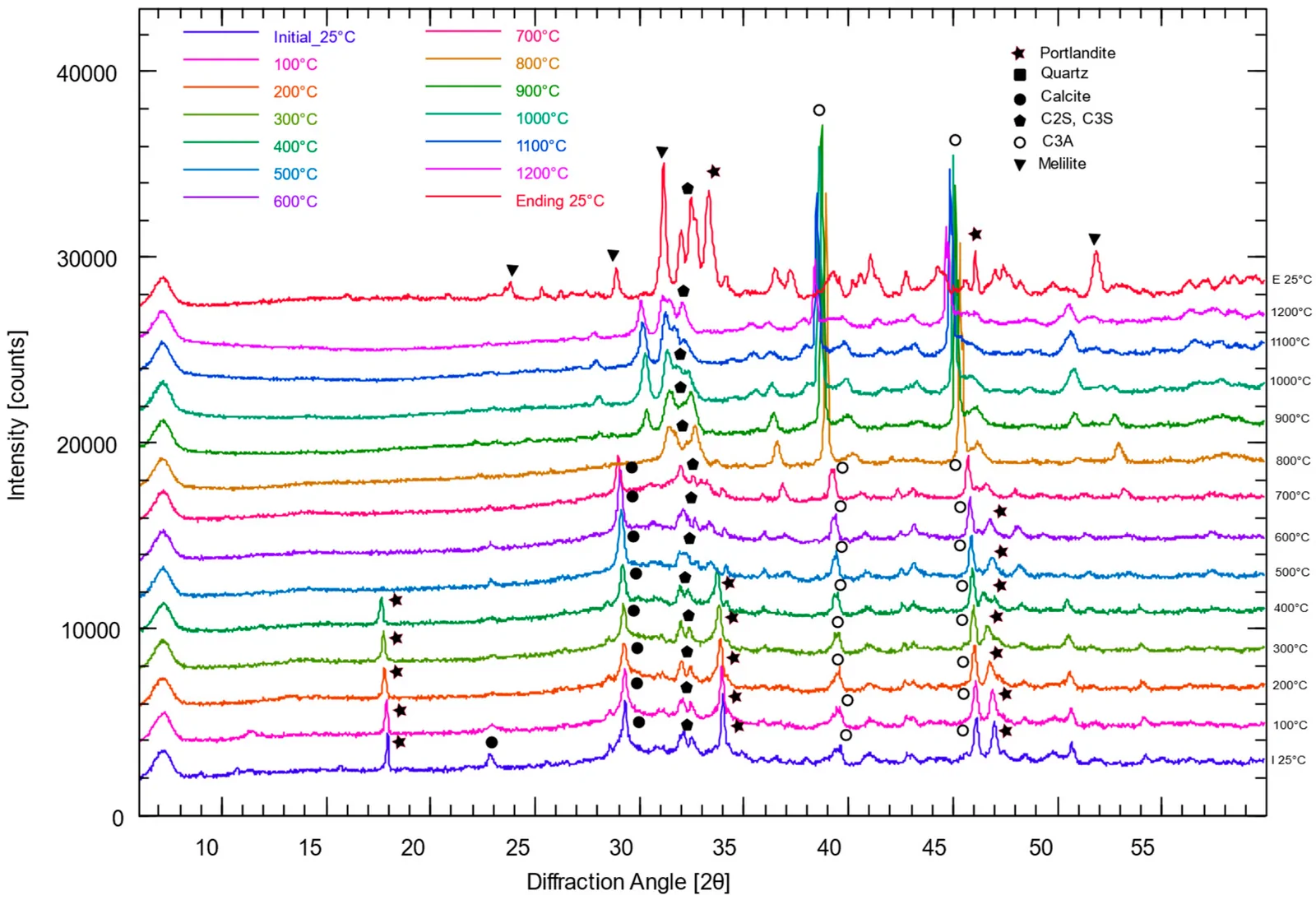
CEM 42.5 GGBFS 50.50 XRD diffractograms. Identified phases: portlandite (★), quartz (■), calcite (⬤), C2S C3S (⬟), C3A (○), melilite (▼).

**Figure 8:**
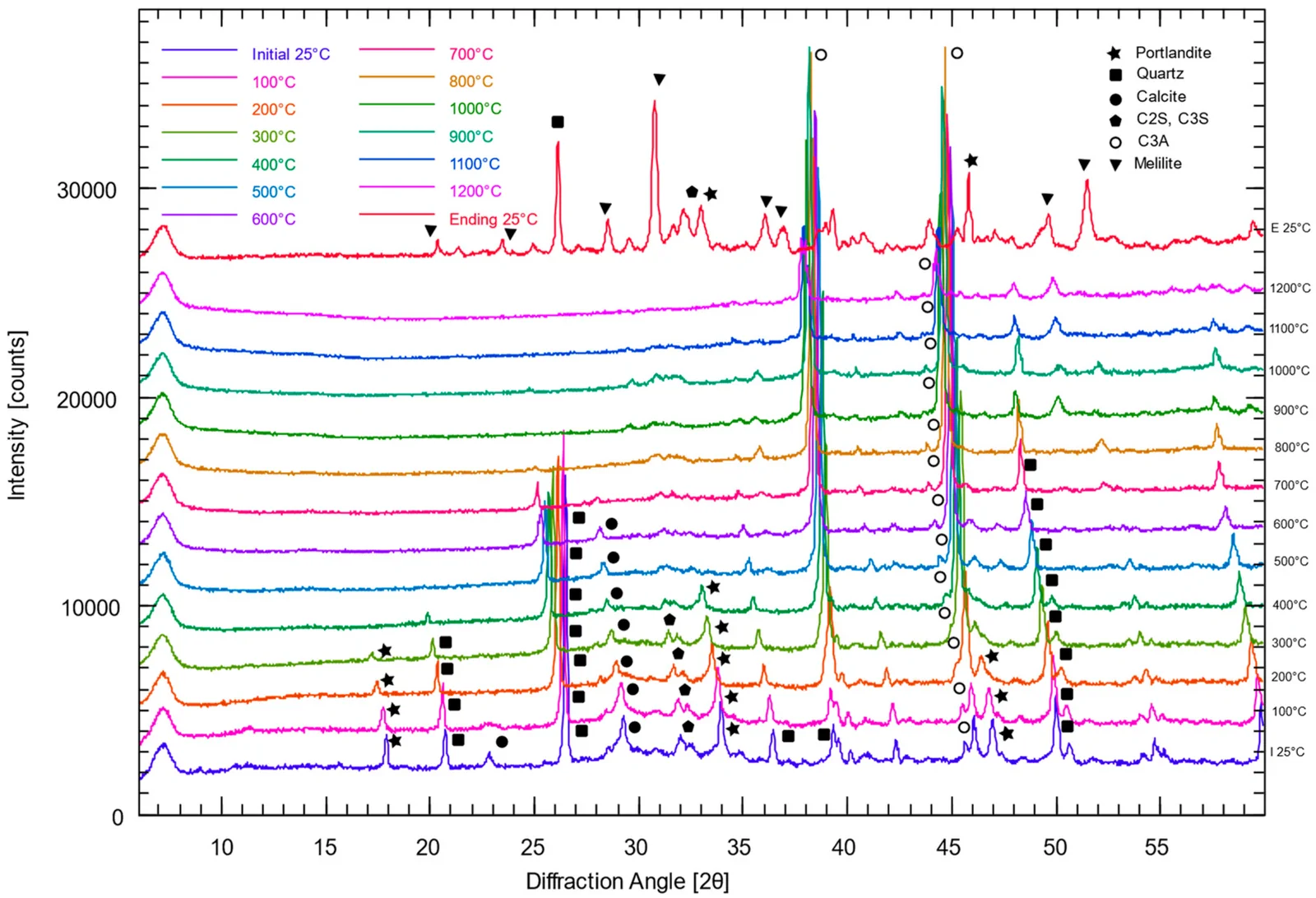
CEM 42.5 GGBFS 50.50 + Q XRD diffractograms. Identified phases: portlandite (★), quartz (■), calcite (⬤), C2S C3S (⬟), C3A (○), melilite (▼).

**Figure 9:**
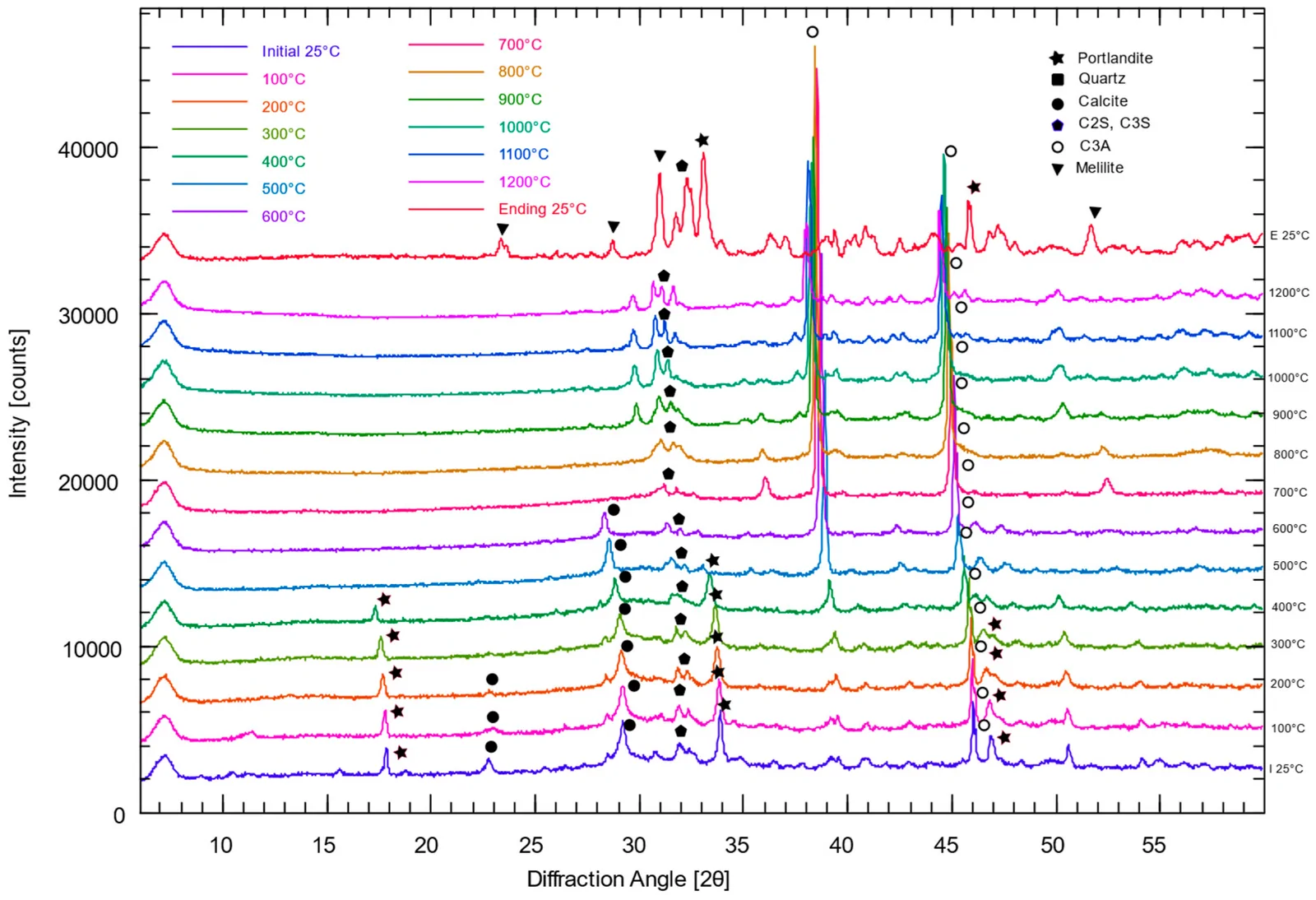
CEM 52.5 GGBFS 50.50 XRD diffractograms. Identified phases: portlandite (★), quartz (■), calcite (⬤), C2S C3S (⬟), C3A (○), melilite (▼).

**Figure 10:**
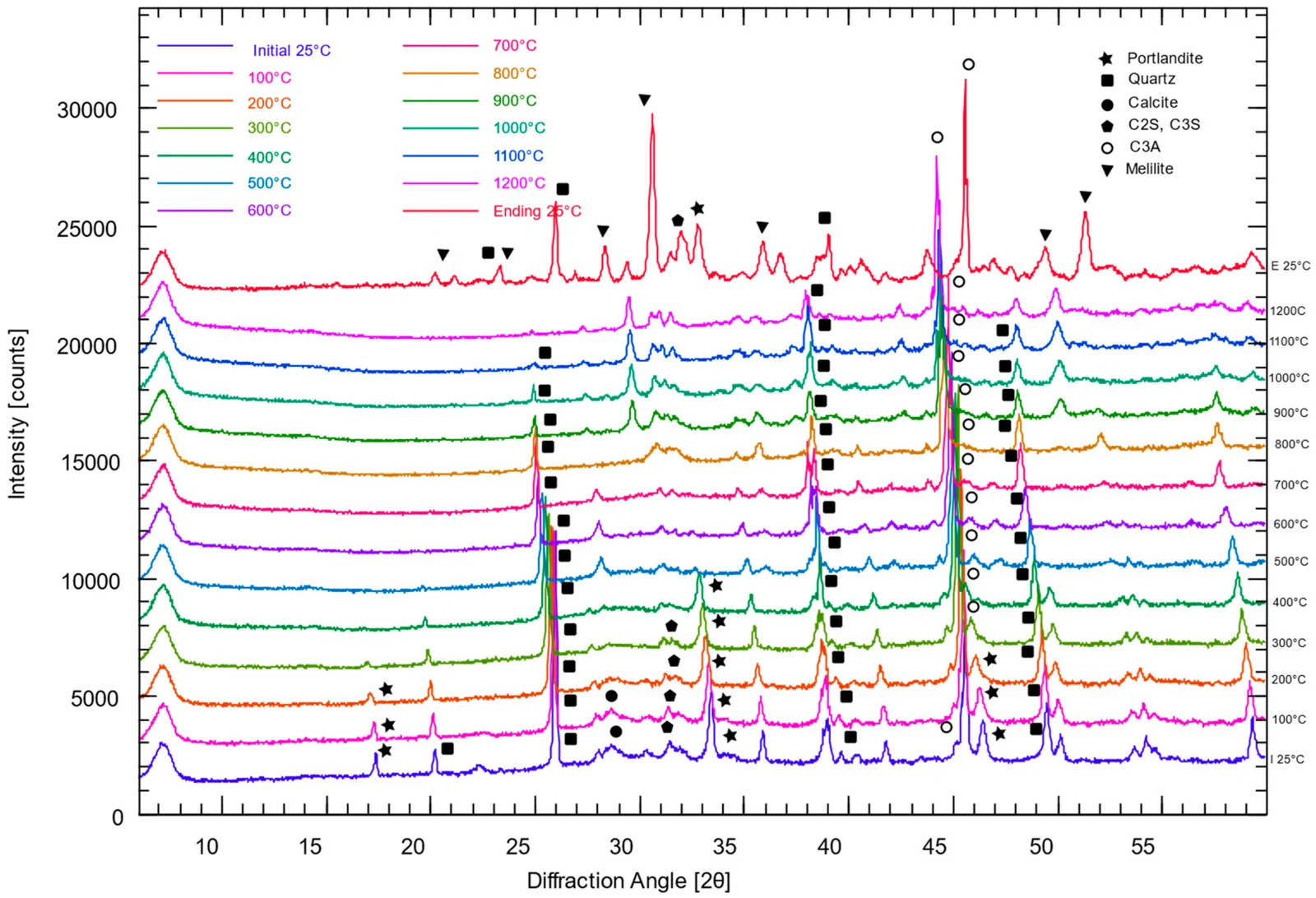
CEM 52.5 GGBFS 50.50 + Q XRD diffractograms. Identified phases: portlandite (★), quartz (■), calcite (⬤), C2S C3S (⬟), C3A (○), melilite (▼).

The X-ray diffractograms are shown in Figure 5, Figure 6, Figure 7, Figure 8, Figure 9 and Figure 10. In general, the intensity (peaks) increased around the 25–35° 2θ range because this is where the main diffraction peaks of the crystalline phases present in the cementitious systems occur. The main observed crystalline phases were portlandite (Ca(OH)_2_) (2θ ≈ 18.1°), calcite (CaCO_3_) (2θ ≈ 29.4°), C_2_S (belite), C_3_S (alite) (all of them mixed at around 2θ ≈ 34.0°), quartz (2θ ≈ 26.6°), and new phases such as melilite (Ca_2_Al(AlSi)O_7_ − Ca_2_MgSi_2_O_7_), (2θ ≈ 33.0°) and tricalcium aluminate (C_3_A) (2θ ≈ 39.0°). The composition was not quantified due to the limitations of the method described in Section 2.2.3.

Due to the complex mineralogy of hydrated blended cements, significant peak overlaps occur in the primary 2θ region (29.0–34.0°). In particular, the main diagnostic reflections of newly forming C_3_A (at 33.2°) and melilite-group phases (at 31.1°) coincide with or lie near the intense diffraction peaks of residual clinker phases (C_3_S at 30.0°, 32.2°, 34.3° and β-C_2_S at 32.1°and 32.6°). To confirm the presence of these phases despite these overlaps, secondary non-overlapping diagnostic reflections (e.g., melilite reflection at 24.0°) were monitored in combination with elemental logic from SEM-EDX microanalysis of ex situ samples.

The shift in the peak positions corresponding to the reflections of each phase at different temperatures is caused by thermal expansion, which leads to changes in the angles between atoms within the material, evident from the changes in d-spacings (XRD peaks moving to lower angle) [27]. The results did not show any significant changes up to 200 °C for samples containing quartz powder (Figure 6, Figure 8 and Figure 10) and 400 °C for mixes containing only cement and GGBFS (Figure 5, Figure 7 and Figure 9). Similar trends were also observed by others. Song et al. [28] reported that the decomposition of cementitious phases occurred at characteristic temperatures, influenced by phase interactions. Ettringite (81–91 °C) and AFm (129–138 °C) dehydrated early, C-S-H lost water between ~80 and 240 °C and began structural breakdown at 615–630 °C, correlating with strength loss above ~620 °C, while hydrogarnet decomposed around 241–244 °C. Ca(OH)_2_ dehydrated between 411 and 427 °C but remained partially stable near 440 °C, and CaCO_3_ decomposed at 648–691 °C, with coexisting phases generally decomposing at lower temperatures than their pure counterparts.

When the paste samples without quartz powder reach temperatures > 600 °C, the calcite peak (CaCO_3_) (2θ ≈ 29.4°) disappeared (Figure 5, Figure 7 and Figure 9); Table 5. At that temperature, it decomposes and releases carbon dioxide.

The decomposition of calcite results in the deposition of CaO, which can lead to increased porosity and potentially reduce the material’s mechanical strength [29] and can react during further heating with silicates present in the cement to form new high-temperature phases as discussed below.

The calcite reflections in samples containing quartz powder (Figure 6, Figure 8 and Figure 10) broaden and drop below the XRD detection limit at temperatures as low as 300 °C. While well-crystallized calcite typically decomposes between 600–800 °C, Igami et al. [30] attributed low-temperature CO_2_ release (300–400 °C) to reactions involving metastable or amorphous carbonate phases. However, the disappearance of diffraction peaks in our XRD data primarily reflects a loss of long-range crystalline order rather than confirmed chemical decomposition, which would require complementary TG/DSC or gas evolution analysis to verify. In these formulations, this earlier decline in reflection intensity is further driven by binder dilution and a higher effective water-to-binder ratio.

A similar trend was observed for portlandite (2θ ≈ 18.1°); Table 6. In samples without quartz (Figure 5, Figure 7 and Figure 9), all peaks are visible up to 400 °C. However, in samples containing quartz (Figure 6, Figure 8 and Figure 10), peaks are only visible up to 200 °C. As the temperature increases, the intensity and integrals of the peaks decrease, indicating gradual decomposition. This behavior is also influenced by the mixtures’ higher water coefficient and lower binder content. Mentioned decomposition releases H_2_O, which can increase porosity and, as a result of the subsequent release of CO_2_ from the calcite, potentially lead to flaking.

Ca(OH)_2_ → CaO + H_2_O, (1)

The sample containing CEM II/B-V 32.5 R and GGBFS (Figure 5) exhibits a progressive reduction in primary reflection intensities starting around 700 °C, followed by the emergence of new high-temperature crystalline peaks by 900 °C. For the corresponding mixture containing quartz powder (Figure 6), a significant attenuation and loss of reflection intensities occur at lower temperatures (around 600 °C); however, well-defined crystalline reflections are fully restored upon cooling to ambient temperature.

In contrast, the sample containing CEM I 42.5 N and GGBFS (Figure 7) remains structurally more stable, with C_3_S and C_2_S reflections remaining discernible up to 1200 °C, alongside the formation of new crystalline phases by 900 °C. In the corresponding quartz-bearing formulation (Figure 8), a marked drop in reflection intensities is observed as early as 500 °C, with sharp crystalline reflections similarly reappearing upon cooling to ambient temperature.

Similarly, the sample containing CEM I 52.5 R and GGBFS (Figure 9) maintains distinct crystalline reflections up to 700 °C before new high-temperature phases begin to form at 900 °C. In the quartz-containing mixture (Figure 10), the attenuation of primary reflections occurs at 500 °C, with reflection intensities recovering notably by 800 °C.

The primary diagnostic reflection of quartz is located at 2θ ≈ 26.6° (Figure 6, Figure 8 and Figure 10). In samples containing GGBFS combined with CEM II/B-V 32.5 R or CEM I 42.5 N, a marked reduction and broadening of this quartz reflection intensity occurs around 700 °C. Upon cooling to ambient temperature, the peak intensity is fully restored. In the mixture containing GGBFS and CEM I 52.5 R, this attenuation of the quartz reflection becomes pronounced at temperatures above 1000 °C. Rather than structural amorphization, this behavior is influenced by thermal effects, structural expansion, micro-cracking, and the displacive α-β quartz polymorphic transition occurring at approximately 573 °C [31,32,33]. α-quartz, stable below this temperature, possesses a trigonal crystal structure, whereas β-quartz, stable above 573 °C, exhibits a hexagonal structure. This polymorphic transformation is rapid and fully reversible, accompanied by significant anisotropic thermal expansion.

In all cases, after cooling, a new phase was observed. Typically, in blast furnace slag that cools slowly, a phase known as melilite forms [34]. Melilite is a solid solution composed of gehlenite (2CaO·Al_2_O_3_·SiO_2_) and akermanite (2CaO·MgO·2SiO_2_), incorporating all the main components of blast furnace slag: CaO, SiO_2_, MgO, and Al_2_O_3_. Since the selected GGBFS is rich in magnesium, akermanite is more likely to form and dominate in this solid solution during crystallization after high-temperature exposure. The presence of akermanite in cement can influence its properties [35]. For example, it can impact the hydration process, the mechanical properties of the hardened cement, and its durability. It helps to maintain the stability of the composite material when exposed to high temperatures. Stronger bonds are less likely to degrade in such conditions. Creating akermanite (Ca_2_MgSi_2_O_7_) from C-A-S-H (calcium aluminate silicate hydrates) involves high-temperature reactions [36]. The synthesis typically occurs through the optimum combination of raw materials containing calcium (calcium carbonate (CaCO_3_) or calcium oxide (CaO)), magnesium (magnesium oxide (MgO)), and silicon (silica (SiO_2_)), and elevated temperatures, typically in the range of 1200–1400 °C, [37]. The high-temperature environment facilitates the solid-state reaction between calcium, magnesium, and silica compounds, leading to the formation of akermanite.

The general reaction to form akermanite can be represented as

2CaO + MgO + 2SiO_2_ → Ca_2_MgSi_2_O_7_, (2)

**Figure 11:**
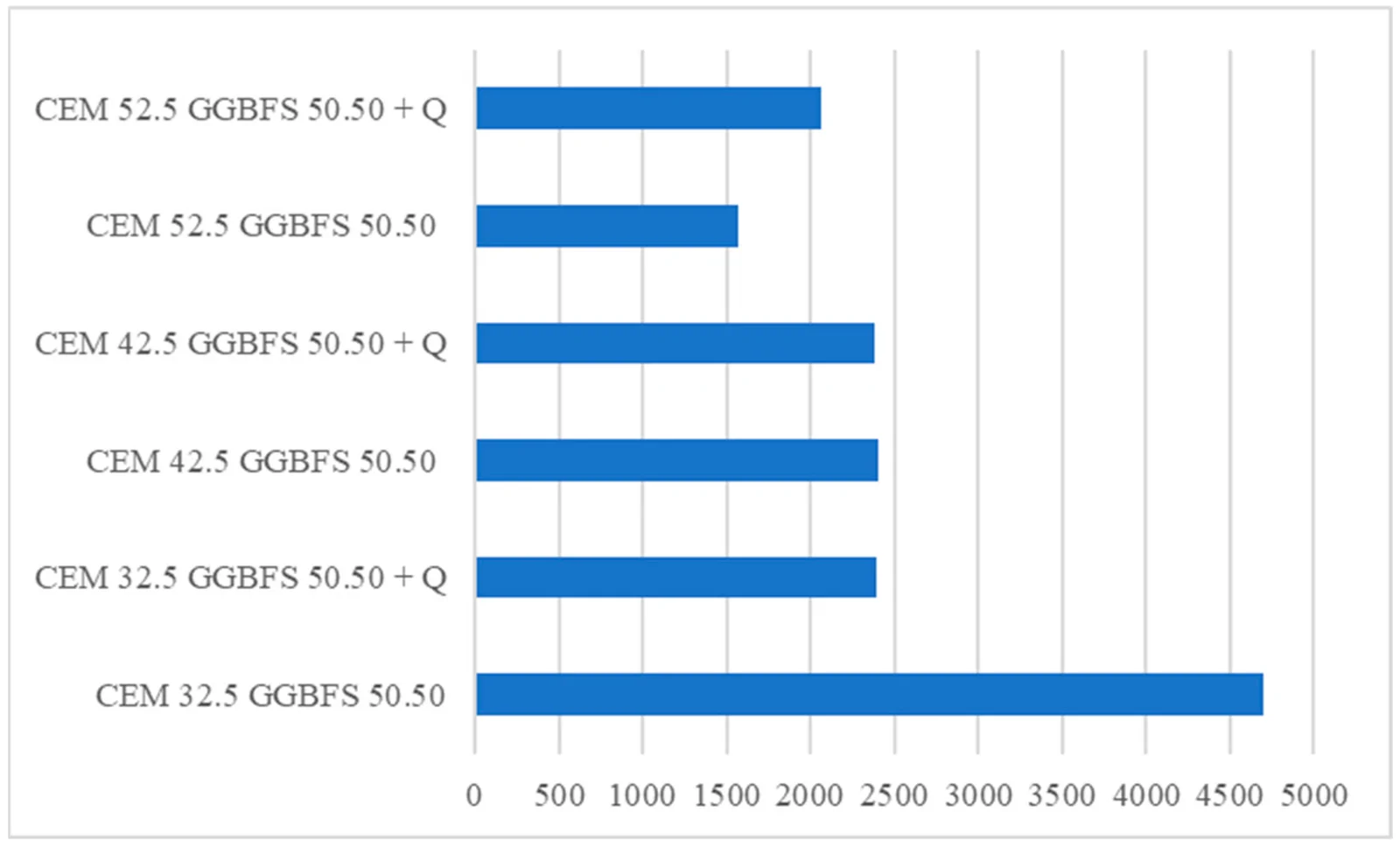
Melilite peak integral (2θ ≈ 33.0°).

The most prominent XRD reflections corresponding to the melilite group were observed in the mix containing CEM II/B-V 32.5 R and GGBFS without quartz powder (Figure 5). Rather than a definitive qualitative XRD assignment, the formation of a melilite-group phase—likely dominated by an akermanite-rich composition—is suggested by the high MgO content of the GGBFS (Table 1) and supported by local SEM-EDX elemental ratios. The presence of fly ash in this cement type provides additional reactive silica (SiO_2_), which further facilitates melilite formation at elevated temperatures. No clear trend regarding melilite content was observed with respect to the addition of quartz powder (Figure 11). The relatively low clinker content in CEM II/B-V 32.5 R results in a limited supply of portlandite, which is shared between the hydration of fly ash and GGBFS. This restricts the initial extent of the GGBFS reaction, leaving a larger fraction of unreacted slag that can subsequently devitrify into melilite during heating.

Akermanite has a high melting point (around 1450 °C) [38], which means it can withstand high temperatures without decomposing or losing its structural integrity. Akermanite has thermal expansion properties compatible with those of the cementitious matrix [39]. This compatibility could possibly reduce internal stress caused by temperature fluctuations, thereby minimizing the risk of thermal cracking. In cases where cracks form, the self-healing properties of akermanite can help seal these cracks, preventing further propagation and maintaining the structural integrity of the concrete.

**Figure 12:**
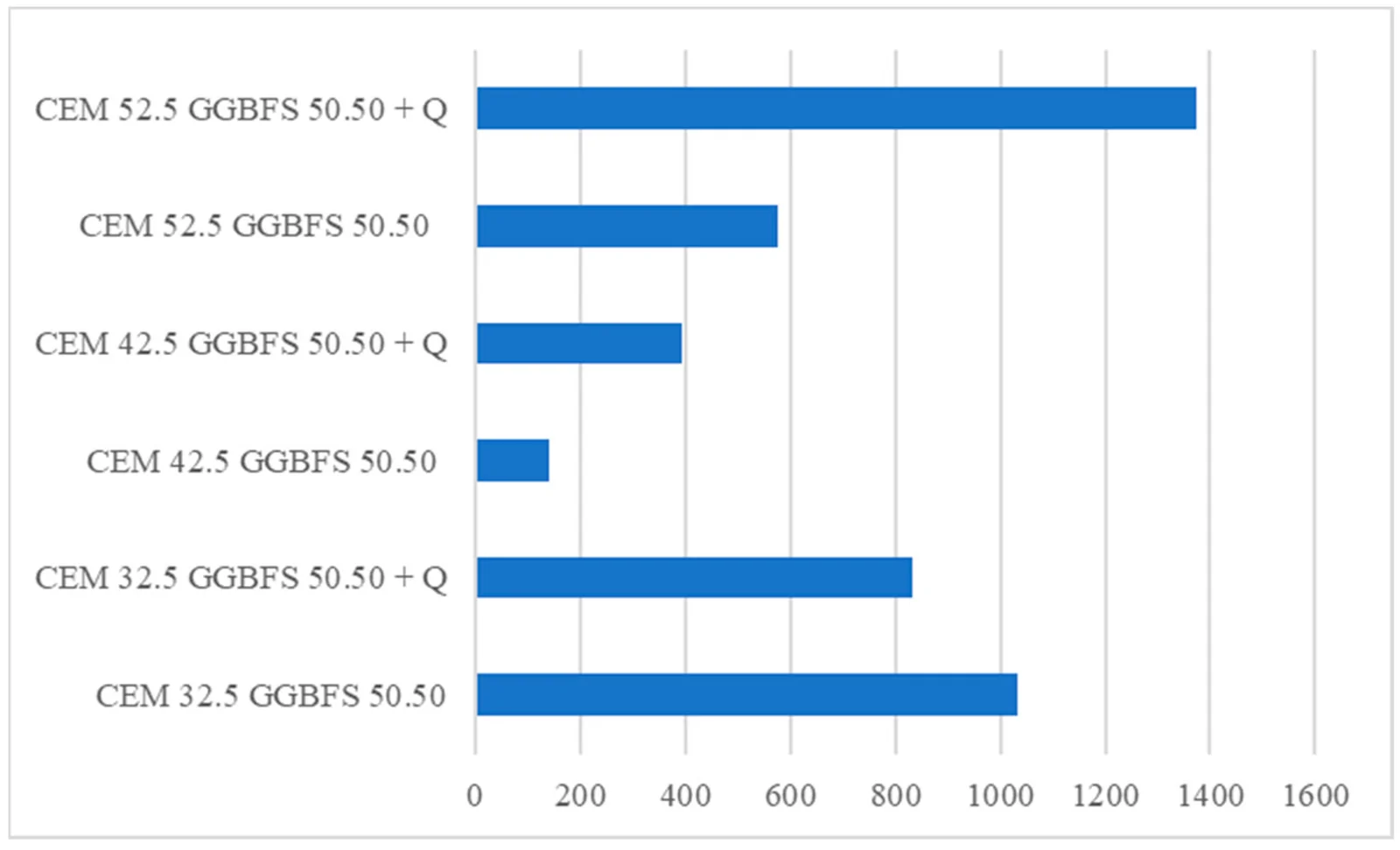
Tricalcium aluminate peak integral (C3A) (2θ ≈ 39.0°).

After exposure to high temperatures and a cooling-down process, tricalcium aluminate (C_3_A) emerged as a new phase in all studied samples (Figure 12). Due to the decomposition of various components in the cement paste, including calcium silicates and aluminates, they decompose and react. The presence of slag, which contains additional alumina (Al_2_O_3_) and silica (SiO_2_), can contribute to these reactions. However, there is no free Al_2_O_3_ in the slag as discrete units since it is entirely bonded into the silicate glass. Tricalcium aluminate (C_3_A) may form through reactions between calcium oxide (CaO) and aluminum oxide (Al_2_O_3_) released during decomposition:

3CaO + Al_2_O_3_ → Ca_3_Al_2_O_6_ (C3A), (3)

There is the possibility that in case of rehydration, C_3_A can react with gypsum (CaSO_4_·2H_2_O) to form ettringite (C_6_A_6_H_32_S_3_), which can potentially contribute to volume expansion and internal stresses, leading to potential cracking [40].

**Figure 13:**
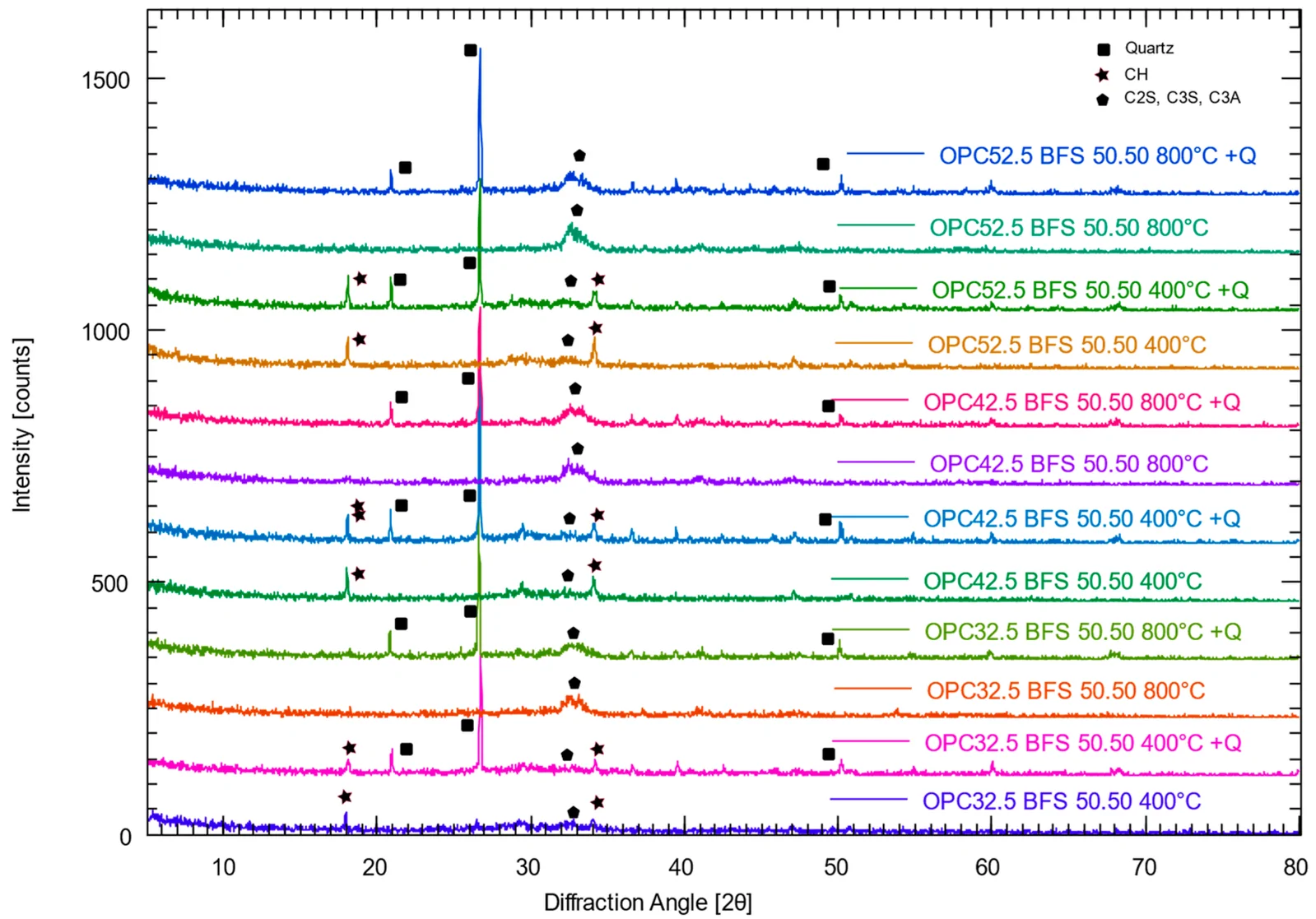
All blends XRD diffractograms. Identified phases: CH (★), quartz (■), C2S C3S C3A (⬟).

The X-ray diffraction (Figure 13) achieved after exposing samples to 400 °C and 800 °C and letting them cool down to ambient temperature did not show significant phase changes.

After exposing samples to 400 °C, typical phases such as calcium silicate hydrates (C-S-H) are still present. The XRD patterns for samples containing quartz powder show characteristic peaks corresponding to quartz, indicating its presence and stability at these temperatures.

At 800 °C, the XRD patterns suggest the early appearance of calcium aluminate phases. These phases are likely to form as the original hydration products decompose or transform due to the elevated temperature. The quartz peaks in the samples with quartz powder remain stable, leading to the conclusion that quartz does not react significantly or decompose at this temperature, thus maintaining its crystalline structure [41,42]. No new additional phases, such as melilite, were found.

The observed phase transformations have direct implications for concrete performance at high temperatures. The thermal dehydration and breakdown of C-S-H and related hydration products reduce the binding capacity, thereby increasing matrix porosity and weakening the microstructure. In quartz-containing formulations, the reversible polymorphic transitions of quartz, combined with differential thermal expansion and higher effective water-to-binder ratios, induce internal stresses that promote microcracking. Meanwhile, the formation of melilite-group phases at elevated temperatures demonstrates the progressive structural reorganization of the silicate network. Together, these microstructural and mineralogical transformations explain the loss of integrity and the reduction in mechanical performance of blended cement-based materials exposed to elevated temperatures [43].

### 3.2. SEM and EDX Analysis

When correlating high-temperature XRD results with SEM observations and porosity measurements, the fundamental differences between in situ and ex situ characterization methods must be considered. In situ XRD directly monitors phase transformations, lattice expansion, and reversible polymorphic transitions (such as the α-β quartz transformation at ~573 °C) at elevated temperatures. In contrast, ex situ SEM microstructural observations and image-based porosity measurements represent the material state after cooling to ambient conditions. During cooling, thermal contraction and differential thermal stresses between distinct phases induce micro-cracking and volumetric changes. Consequently, while ex situ SEM and porosity capture the permanent thermal damage and retained high-temperature phases (such as melilite), they also incorporate cooling-induced artifacts that must be distinguished from the in situ thermal state.

The data obtained from scanning electron microscopy (SEM) paired with energy dispersive X-ray spectroscopy (EDX) are shown in Table 7. For the mixture CEM 32.5 GGBFS 50.50 without quartz powder, aluminium, silicon, and calcium levels generally decreased with increasing temperature. The Si/Ca atomic ratio slightly increased at 400 °C before decreasing, and both the Al/Ca and Al/Si ratios remain relatively stable across the temperature range.

When quartz powder was added to CEM 32.5 GGBFS 50.50, the trends changed slightly. Aluminium and silicon contents increased at 400 °C, decreased at 800 °C, and rose again at 1200 °C. Calcium decreased initially but increased at 1200 °C. The Si/Ca ratio tended to increase with the temperature, indicating a stabilizing effect of the quartz powder. The Al/Ca atomic ratio remained constant but then increased at 1200 °C. The Al/Si atomic ratio decreased initially but stabilized at higher temperatures.

For CEM 42.5 GGBFS 50.50 without quartz powder, the aluminium amount decreased until 800 °C and then increased at 1200 °C. Silicon and calcium showed similar trends, decreasing until 800 °C and increasing at 1200 °C. The Si/Ca ratio remained relatively stable, while the Al/Ca and Al/Si ratios exhibited only minor changes.

With the addition of quartz powder to CEM 42.5 GGBFS 50.50, both aluminium and silicon contents decreased at 400 °C and 800 °C but increased at 1200 °C. Calcium also decreased until 800 °C and then increased at 1200 °C. The Si/Ca ratio increased steadily with the temperature, while the Al/Ca and Al/Si ratios remained relatively stable, showing only a slight increase at higher temperatures.

For CEM 52.5 GGBFS 50.50 without quartz powder, aluminium content decreased at 400 °C but increased at higher temperatures. Silicon content increased at 800 °C before stabilizing, while calcium content decreased at 400 °C, increased at 800 °C, and then decreased again at 1200 °C. The Si/Ca and Al/Ca ratios decreased initially before increasing, while the Al/Si ratio followed a similar trend.

When quartz powder is added to CEM 52.5 GGBFS 50.50, aluminium and silicon contents decrease at 400 °C and 800 °C but increase at 1200 °C. Calcium content shows a decreasing trend until 800 °C, followed by an increase at 1200 °C. The Si/Ca ratio increases at 400 °C, decreases at 800 °C, and then increases again at 1200 °C. The Al/Ca and Al/Si ratios remain relatively constant across the temperature range.

The SEM-EDX analysis confirms that quartz powder influences the elemental composition of mixes with the temperature. For mixes without quartz, aluminium, silicon, and calcium generally decreased with increasing the temperature, with minor variations in atomic ratios. When quartz was added, silicon and aluminium levels tended to be maintained or recover at higher temperatures (800–1200 °C), and the Si/Ca atomic ratio generally increased, indicating enhanced thermal stability. These compositional trends help explain the stabilizing effect observed in XRD measurements, as quartz preserves silicon-rich phases and promotes the formation of calcium–aluminate–silicate phases, reducing microstructural degradation, limiting porosity growth, and maintaining mechanical integrity at elevated temperatures.

Supporting these findings, a recent study [44] utilizing high-throughput atomistic modelling to investigate the structural and mechanical properties of calcium aluminate silicate hydrate (C-A-S-H) across a range of Ca/Si and Al/Si ratios found that increasing the Al/Si ratio promotes chain polymerization. It is leading to longer mean chain lengths and improved mechanical performance. This aligns with earlier work by L’Hopital et al. [45], who demonstrated that Al incorporation into C–S–H strongly depends on the Ca/Si ratio, producing distinct structural configurations (C–S–Ha at low Ca/Si and C–S–Hb at high Ca/Si). At low Ca/Si ratios, Al preferentially substitutes Si in non-bridging sites, whereas at higher Ca/Si ratios substitution shifts to bridging positions, promoting chain rupture and reorganization. These insights suggest that quartz powder, by supplying additional silicon and promoting higher Al uptake, contributes to stronger polymerized C-A-S-H networks, consistent with the stabilizing trends observed in SEM-EDX analysis.

However, mixtures containing quartz display a dual response: while supporting silicon-rich phase formation, the combined effects of the α-β quartz transition, binder dilution, and a higher effective water-to-binder ratio induce internal stresses and cause earlier reductions in portlandite and calcite XRD reflection intensities, which may theoretically affect high-temperature mechanical performance.

### 3.3. Porosity

**Figure 14:**
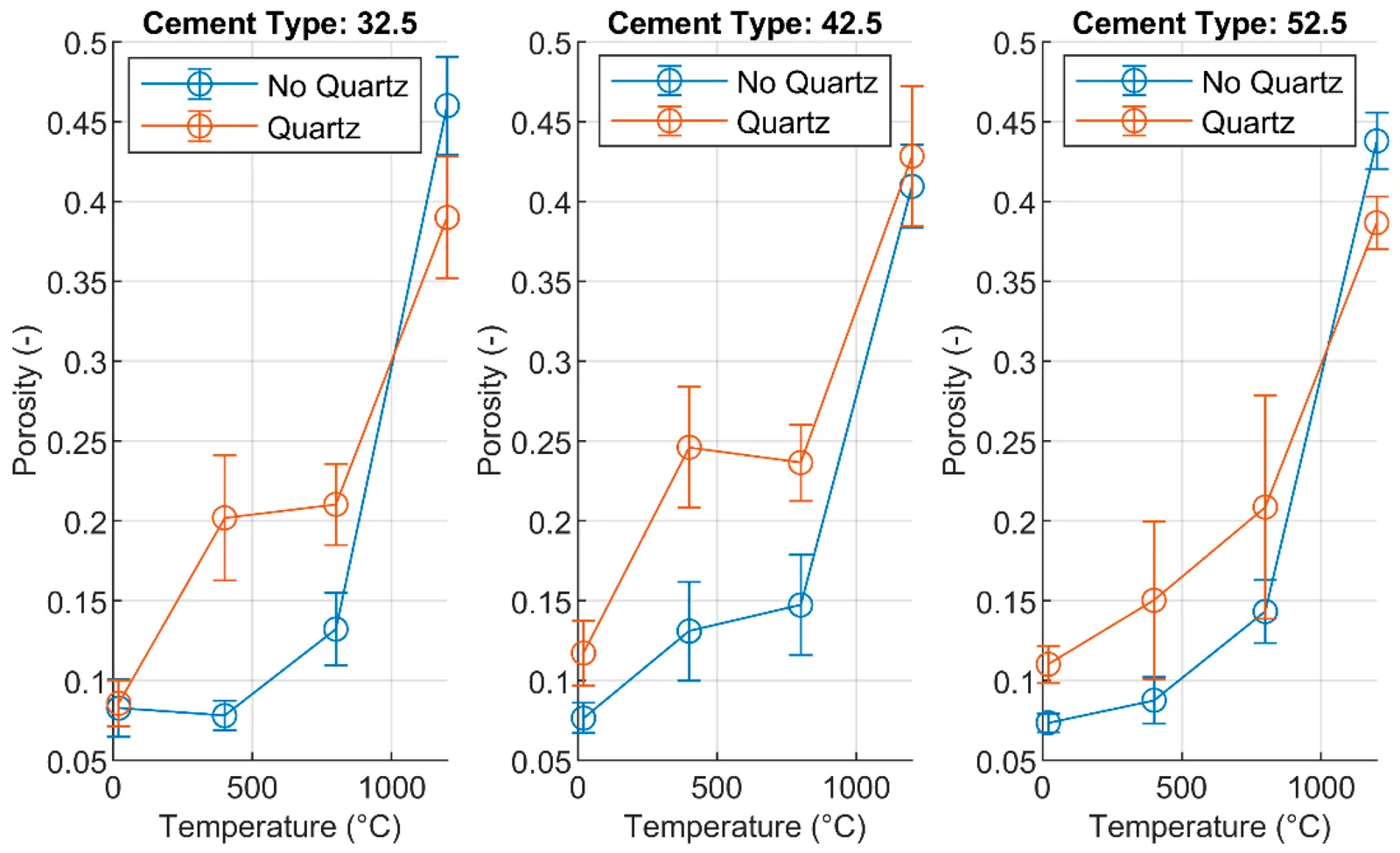
Results for different types of cement with and without quartz addition vs. temperature load.

An analysis of matrix porosity across the studied mixtures revealed clear trends depending on cement type, quartz addition, and exposure temperature. Overall, the incorporation of quartz powder caused a substantial shift toward higher matrix porosity across all evaluated conditions. Specifically, samples containing quartz exhibited visibly higher porosity compared to their quartz-free counterparts already at an ambient temperature (Figure 14). This baseline increase can be attributed to the dilution effect and the effective increase in the water-to-binder ratio resulting from quartz replacement.

Among the evaluated binders, mixtures prepared with CEM I 42.5 N consistently displayed higher mean porosity values than those made with CEM II/B-V 32.5 or CEM I 52.5 R, whereas CEM II/B-V 32.5 R and CEM I 52.5 R showed closely comparable porosity levels across most temperature states. As expected, thermal exposure to 400 °C, 800 °C, and 1200 °C progressively increased the mean porosity across all mixtures compared to the reference state (20 °C). In mixtures containing quartz powder, porosity rose sharply between ambient conditions and 400 °C for CEM II/B-V 32.5 R and CEM I 42.5 N, followed by a plateau between 400 °C and 800 °C. This plateau indicates a temporary stabilization of pore structure development in this intermediate temperature range. Conversely, in the absence of quartz, CEM II/B-V 32.5 R and CEM I 42.5 N exhibited only a minor increase in porosity up to 400 °C, followed by a steep increase between 400 °C and 800 °C.

At the maximum thermal load of 1200 °C, the porosity difference between quartz-bearing and quartz-free samples narrowed significantly for all cement types. This suggests that at extreme temperatures, widespread thermal damage, phase melting, and microstructural restructuring become the dominant factors governing total porosity, diminishing the relative impact of initial quartz addition.

## 4. Conclusions

Thermal stability of cementitious systems is strongly influenced by cement type, ground granulated blast furnace slag, and the presence of quartz powder. Optimizing the composition of the binder and the silica content makes it possible to control phase development at high temperatures and the porosity of the matrix, thereby offering a means of adjusting the thermal stability of the microstructure of blended cement systems.

- Addition of quartz exhibits a dual effect on cementitious systems.
  It provides additional silicon that helps maintain silicon-rich phases and supports the formation of calcium–aluminate–silicate structures, enhancing microstructural stability at high temperatures.
  In quartz-containing mixtures, the reduction of portlandite (Ca(OH)_2_) and calcite (CaCO_3_) XRD reflections occurs at lower temperatures, driven by binder dilution and an altered effective water-to-binder ratio.
  Mixes without quartz exhibit greater mineralogical stability at intermediate temperatures.
- Quartz-containing mixtures exhibit earlier reductions in characteristic XRD reflection intensities and develop higher matrix porosity in the 400–800 °C range—driven by the combined effects of the α-β quartz transition, binder dilution, and an increased effective water-to-binder ratio—though these microstructural differences diminish at temperatures above 1200 °C.
- Higher alumina, silica, and fly ash content in CEM II/B-V 32.5 R promotes the formation of calcium–aluminate–silicate phases. Melilite forms as a new crystalline phase after heating, influenced by cement composition and the presence unreacted or partially hydrated clinker.
- Further research is recommended to investigate the mechanical impact of increased matrix porosity and phase alterations in quartz-containing formulations, specifically examining to what extent high-temperature melilite and C_3_A formation can stabilize the thermally damaged matrix.

## Figures and Tables

**Table 1:** Physical and chemical properties of cements (data provided by producers).

	CEM II/B-V 32.5 R—HSR	CEM I 42.5 N	CEM I 52.5 R
Blaine fineness (m^2^/kg)	352	330	362
Setting time (min)	298	170	223
LOI (%)	n/a	0.4–0.5	2.56
Alkali, Na_2_Oeq (%)	1.30	0.4–0.58	0.62
SO_3_ (%)	2.64	-	<4.0
Fly ash (%)	29.3	-	-

**Table 2:** Chemical composition of GGBFS Merit, as determined by XRF (information provided by the manufacturer).

CaO	SiO_2_	Al_2_O_3_	Fe_2_O_3_	MgO	Na_2_O	K_2_O	TiO_2_	MnO	SO_3_	LOI
30.4	35.0	14.3	0.3	16.1	0.6	0.7	2.8	0.5	0.7	0.9

**Table 3:** Chemical profile of Norquartz 45.

Component	SiO_2_ (wt.%)	Al_2_O_3_ (wt.%)	Fe_2_O_3_ (wt.%)	LOI	pH	% Passing 45 µm	Density (g/cm^3^)
Average (%)	99.6	0.25	0.02	0.15	6.5	99.2	2.65

**Table 4:** Raw materials mixing ratio.

	Cement (g)	GGBFS (g)	Quartz (g)	Water (g)
Set 1	40	40	-	32
Set 2	32	32	16	32

**Table 5:** Calcite peak integral (2θ ≈ 29.4°).

Temp. (°C)	Amb.	100	200	300	400	500	600	700
CEM 32.5 GGBFS 50.50	651	639	522	459	441	612	389	-
CEM 32.5 GGBFS 50.50 + Q *	-	-	-	-	-	-	-	
CEM 42.5 GGBFS 50.50	762	749	633	569	631	1069	1074	512
CEM 42.5 GGBFS 50.50 + Q	456	348	311	-	-	-	-	-
CEM 52.5 GGBFS 50.50	654	810	672	635	602	522	365	-
CEM 52.5 GGBFS 50.50 + Q *	-	-	-	-	-	-	-	

* Peak quantification was not possible for the 32.5 and 52.5 samples containing quartz powder due to issues with peak overlap.

**Table 6:** Portlandite peak integral (2θ ≈ 18.1°).

Temp. (°C)	Amb.	100	200	300	400
CEM 32.5 GGBFS 50.50	237	232	203	202	192
CEM 32.5 GGBFS 50.50 + Q	146	128	94	-	-
CEM 42.5 GGBFS 50.50	264	243	242	233	197
CEM 42.5 GGBFS 50.50 + Q	213	177	97	58	-
CEM 52.5 GGBFS 50.50	238	236	211	202	154
CEM 52.5 GGBFS 50.50 + Q	131	103	86	27	-

**Table 7:** Average elements point content SEM-EDX in wt.%.

MIX	Temp.	Al	Si	Ca	Si/Ca	Al/Ca	Al/Si
CEM 32.5 GGBFS 50.50	Ambient	2.00	5.63	10.82	0.53	0.18	0.35
	400 °C	1.71	5.43	9.41	0.58	0.18	0.31
	800 °C	1.42	4.13	9.18	0.45	0.16	0.34
	1200 °C	1.45	4.20	9.30	0.45	0.16	0.34
CEM 32.5 GGBFS 50.50 + Q	Ambient	1.89	7.40	10.35	0.72	0.18	0.26
	400 °C	1.85	7.70	9.68	0.80	0.19	0.24
	800 °C	1.06	4.21	6.94	0.61	0.15	0.25
	1200 °C	2.32	8.01	8.60	0.93	0.27	0.29
CEM 42.5 GGBFS 50.50	Ambient	1.54	6.18	12.26	0.50	0.13	0.25
	400 °C	1.28	5.40	10.25	0.53	0.16	0.24
	800 °C	0.94	3.95	9.47	0.42	0.1	0.24
	1200 °C	1.46	5.80	12.31	0.47	0.12	0.25
CEM 42.5 GGBFS 50.50 + Q	Ambient	1.30	7.71	11.69	0.66	0.11	0.17
	400 °C	1.18	6.52	9.47	0.69	0.13	0.18
	800 °C	0.95	5.42	8.47	0.64	0.11	0.17
	1200 °C	1.75	7.48	9.38	0.80	0.19	0.23
CEM 52.5 GGBFS 50.50	Ambient	1.12	1.72	4.13	0.42	0.27	0.65
	400 °C	0.72	1.33	1.40	0.95	0.51	0.54
	800 °C	1.37	4.62	12.24	0.38	0.11	0.30
	1200 °C	2.26	4.64	10.19	0.46	0.22	0.49
CEM 52.5 GGBFS 50.50 + Q	Ambient	1.70	7.90	12.57	0.63	0.14	0.21
	400 °C	1.36	6.50	9.80	0.66	0.14	0.21
	800 °C	1.25	5.62	9.46	0.59	0.13	0.22
	1200 °C	1.50	7.32	9.60	0.76	0.16	0.21

## Data Availability

The original contributions presented in this study are included in the article. Further inquiries can be directed to the corresponding author.
